# Cathepsin D protects colorectal cancer cells from acetate-induced apoptosis through autophagy-independent degradation of damaged mitochondria

**DOI:** 10.1038/cddis.2015.157

**Published:** 2015-06-18

**Authors:** C S F Oliveira, H Pereira, S Alves, L Castro, F Baltazar, S R Chaves, A Preto, M Côrte-Real

**Affiliations:** 1 Department of Biology, CBMA – Centre of Molecular and Environmental Biology, University of Minho, Campus de Gualtar, Braga, Portugal; 2 ICBAS – Institute of Biomedical Sciences Abel Salazar, University of Porto, Porto, Portugal; 3 Life and Health Sciences Research Institute (ICVS), School of Health Sciences, University of Minho, Braga, Portugal; 4 ICVS/3B's - PT Government Associate Laboratory, Braga/Guimarães, Portugal

## Abstract

Acetate is a short-chain fatty acid secreted by Propionibacteria from the human intestine, known to induce mitochondrial apoptotic death in colorectal cancer (CRC) cells. We previously established that acetate also induces lysosome membrane permeabilization in CRC cells, associated with release of the lysosomal protease cathepsin D (CatD), which has a well-established role in the mitochondrial apoptotic cascade. Unexpectedly, we showed that CatD has an antiapoptotic role in this process, as pepstatin A (a CatD inhibitor) increased acetate-induced apoptosis. These results mimicked our previous data in the yeast system showing that acetic acid activates a mitochondria-dependent apoptosis process associated with vacuolar membrane permeabilization and release of the vacuolar protease Pep4p, ortholog of mammalian CatD. Indeed, this protease was required for cell survival in a manner dependent on its catalytic activity and for efficient mitochondrial degradation independently of autophagy. In this study, we therefore assessed the role of CatD in acetate-induced mitochondrial alterations. We found that, similar to acetic acid in yeast, acetate-induced apoptosis is not associated with autophagy induction in CRC cells. Moreover, inhibition of CatD with small interfering RNA or pepstatin A enhanced apoptosis associated with higher mitochondrial dysfunction and increased mitochondrial mass. This effect seems to be specific, as inhibition of CatB and CatL with E-64d had no effect, nor were these proteases significantly released to the cytosol during acetate-induced apoptosis. Using yeast cells, we further show that the role of Pep4p in mitochondrial degradation depends on its protease activity and is complemented by CatD, indicating that this mechanism is conserved. In summary, the clues provided by the yeast model unveiled a novel CatD function in the degradation of damaged mitochondria when autophagy is impaired, which protects CRC cells from acetate-induced apoptosis. CatD inhibitors could therefore enhance acetate-mediated cancer cell death, presenting a novel strategy for prevention or therapy of CRC.

Colorectal cancer (CRC) is one of the most common cancers worldwide.^1, 2^ In Europe, it is the most diagnosed malignancy and the second cause of cancer mortality in both genders,^2^ highlighting the need for novel strategies to prevent and treat CRC. Short-chain fatty acids (SCFA), namely butyrate, propionate and acetate, are the major by-products of anaerobic bacterial fermentation of undigested fibers in the human colon. As they were reported as antiproliferative and antineoplastic agents that induce differentiation, growth arrest and apoptosis in CRC cell lines,^3, 4, 5, 6^ there has been increased interest in exploiting these natural products in CRC prevention and therapy. The antitumor effect of SCFAs stems from their ability to induce cell death involving mitochondria-mediated apoptosis (caspase-dependent/independent) or necrosis in colon cancer cells.^3, 4, 6^ We also previously implicated another organelle in acetate-induced apoptosis, the lysosome. Indeed, lysosomal membrane permeabilization (LMP) and release of cathepsins into the cytosol can initiate the lysosomal apoptotic pathway either in a mitochondria-independent manner or mediated by mitochondrial destabilization with subsequent release of apoptotic factors.^7, 8^ Among the cathepsins released by LMP, cathepsin D (CatD), originally considered a ‘housekeeping enzyme'^9^ necessary for autophagy^10^ can act as an antiapoptotic or proapoptotic mediator depending on the cell type and context.^10, 11, 12^ However, the exact mechanisms triggered by CatD following LMP in cancer cells, as well as the signaling to and/or from mitochondria, remain to be clarified.

In a previous study, we demonstrated that CatD is released into the cytosol and protects cells undergoing acetate-induced apoptosis.^5^ These results were in agreement with our data showing that Pep4p, the yeast ortholog of human CatD, translocates from the vacuole to the cytosol during mitochondria-mediated acetic acid-induced apoptosis in *Saccharomyces cerevisiae*,^13^ and that it has a protective role in this process dependent on its catalytic activity.^14^ However, the mechanisms by which CatD protects CRC cells from acetate exposure are still unknown. It had been shown that SCFAs (namely butyrate and propionate) induce autophagy in CRC cells, which serves as an adaptive strategy to delay mitochondria-mediated apoptotic cell death.^15, 16, 17^ In contrast, we showed that yeast CatD is involved in acetic acid-induced mitochondrial degradation independently of autophagy.^13^ We therefore hypothesized the same would occur in the response of CRC cells to acetate. In this work, we assessed how CatD affects the mitochondrial alterations induced by acetate in CRC cell lines, and specifically evaluated its role in mitochondrial degradation. Our results indicate that the antineoplastic mechanism of acetate is characterized by an accumulation of reactive oxygen species (ROS) and changes in mitochondrial mass and mitochondrial membrane potential (ΔΨm). We further show that CatD, but not CatB or CatL, is involved in the degradation of the dysfunctional mitochondria to enhance CRC cell survival in response to acetate, which impairs autophagy. Our data therefore indicate that acetate-induced apoptosis involves cross-talk between the lysosome and mitochondria, and support using probiotics and inhibiting CatD as a prevention or therapeutic strategy in CRC.

## Results

### Acetate leads to oxidative stress, changes in ΔΨm and increased mitochondrial mass in CRC cells

**Figure 1:**
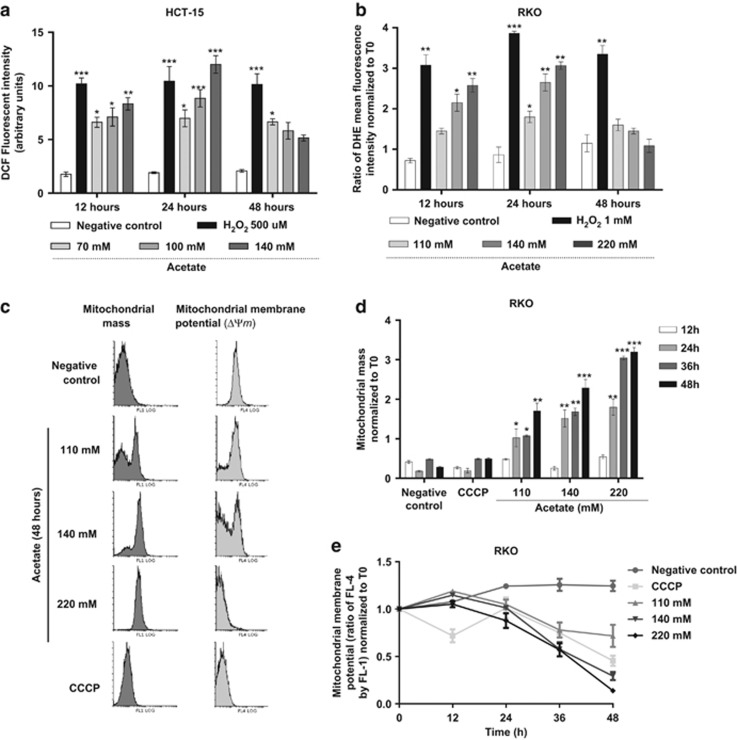
Acetate induces oxidative stress and mitochondrial dysfunction in CRC cells. (**a** and **b**) HCT-15 and RKO cells were incubated with acetate (70, 100 or 140 mM for HCT-15 cells; 110, 140 or 220 mM for RKO cells) for 12, 24 and 48 h. HCT-15- and RKO-negative control cells were incubated with fresh complete medium for the same times, whereas positive control cells were incubated with 500 *μ*M and 1 mM H_2_O_2_, respectively. (**a**) Accumulation of H_2_O_2_ in HCT-15 cells was detected with H_2_DCF-DA using a fluorescence microplate reader. (**b**) Accumulation of superoxide anion (O_2_^−^) in RKO cells was detected with DHE and quantified by flow cytometry. (**c**–**e**) Detection of mitochondrial dysfunction in RKO cells using MitoTracker Green FM and MitoTracker Red CMXROs double staining. Cells were incubated with 110, 140 or 220 mM of acetate for up to 48 h and then stained with 400 nM MitoTracker Green and 200 nM MitoTracker Red for 30 min at 37 ºC in the dark. (**c**) Representative histograms of mitochondrial dysfunction in cell exposed to 110, 140 or 220 mM of acetate for 48 h. There was a significant increase in the green mean fluorescence intensity (MitoTracker Green), indicative of an increase in mitochondrial mass, and a decrease in the mean red fluorescence intensity (MitoTracker Red CMXRos), indicative of changes in the ΔΨm. (**d**) Acetate treatment induces an increase in the mean green fluorescence intensity normalized to the green mean fluorescence intensity at time 0 (*T*0), indicating an increase in mitochondrial mass. (**e**) Acetate treatment induces an alteration in the ΔΨm normalized to mitochondrial mass, assessed by the ratio between the mean red fluorescence intensity (FL-4) and the mean green fluorescence intensity (Fl-1) normalized to *T*0. For each time point, the values of at least three independent experiments are represented. Bonferroni's test *P≤0.05; **P≤0.01 and ***P≤0.001 compared with negative control cells. In (**c**–**e**), cells were incubated with fresh complete medium or with 50 *μ*M CCCP, which were used as a negative and positive control, respectively

Acetate-induced apoptosis has been associated with mitochondrial dysfunction, including increased ROS and ΔΨm alterations, although only in HT-29 cells.^3, 4^ We also previously showed that CRC-derived cells HCT-15 and RKO exposed to physiological concentrations of acetate exhibit characteristic apoptotic markers.^5^ However, an effect on mitochondrial mass, as suggested by our studies in yeast,^13^ has never been described. We therefore sought to determine comprehensively how acetate affects mitochondrial function in HCT-15 and RKO cells, in particular ROS accumulation and changes in ΔΨm and mitochondrial mass. For this purpose, cells exposed to different concentrations of acetate were stained with the ROS probes 2,7-dihydrodichlorofluorescein diacetate (H_2_DCF-DA) or dihydroethidium (DHE), to detect hydrogen peroxide (H_2_O_2_) or superoxide anion (O_2_^−^), respectively. ROS levels in HCT-15 and RKO cells consistently increased in response to acetate in a dose-dependent manner after 12 and 24 h of exposure, and then slightly decreased after 48 h (H_2_O_2_ in HCT-15 (Figure 1a) and RKO cells (not shown), and O_2_^−^ in RKO cells (Figure 1b)). To determine if the acetate-induced ROS accumulation could be due to inhibition of cellular antioxidant defenses, we used a qualitative assay to measure catalase activity.^18^ We found that exposure to a half-maximal inhibitory concentration (IC_50_) of acetate slightly decreased catalase activity in HCT-15 and RKO cells, although more evidently in the latter (Supplementary Figure S1).

In addition to resulting from a decrease in antioxidant defenses, ROS accumulation can be an early event of the apoptotic process, associated with other mitochondrial dysfunctions.^19^ We therefore assessed if acetate induces further mitochondrial alterations in CRC cells. We found that acetate, similar to CCCP (carbonyl cyanide *m*-chlorophenylhydrazone), decreased the ΔΨm, as it lowered mitochondrial accumulation of DiOC_6_(3) and of MitoTracker Red CMXRos in RKO cells and of DiOC_6_(3) in HCT-15 cells (Supplementary Figure S2), while increasing cytosolic fluorescence of the probes. As changes in ΔΨm can result from changes in mitochondrial mass, we quantitatively assessed these two parameters simultaneously in RKO cells stained with both MitoTracker Red CMXRos and MitoTracker Green FM, by flow cytometry. In addition to a decrease in the mean red fluorescence, indicative of decreased ΔΨm (Figure 1c), acetate increased the mean green fluorescence, indicative of an increase in mitochondrial mass (Figures 1c and d). After normalizing acetate-induced changes in ΔΨm to mitochondrial mass, and in relation to time zero (Figure 1e), it became evident that a short exposure to acetate induces a small increase in ΔΨm (Figure 1e). This hyperpolarization was apparent after 12 h of exposure to 110 and 140 mM but not to 220 mM acetate. For longer exposure times, this ratio significantly decreased for all concentrations, indicative of ΔΨm dissipation (as verified for the control CCCP treatment). We also investigated the effect of acetate on the levels of mitochondrial proteins, namely the apoptosis-inducing factor (AIF), the voltage dependent anion channel (VDAC1) and a subunit of the outer mitochondrial membrane translocator (TOM22). We found all doses of acetate, as well as etoposide, increased the levels of AIF and VDAC1 in RKO and, to a higher extent, in HCT-15 cells (Supplementary Figure S3). In addition, acetate, but not etoposide, led to increased levels of TOM22.

Our results indicate that acetate causes mitochondrial dysfunction, increasing ROS accumulation, perturbing ΔΨm and increasing mitochondrial mass in a time- and dose-dependent manner, suggesting that acetate impairs the turnover of harmful mitochondria.

### Acetate inhibits basal autophagy and impairs autophagy induction

**Figure 2:**
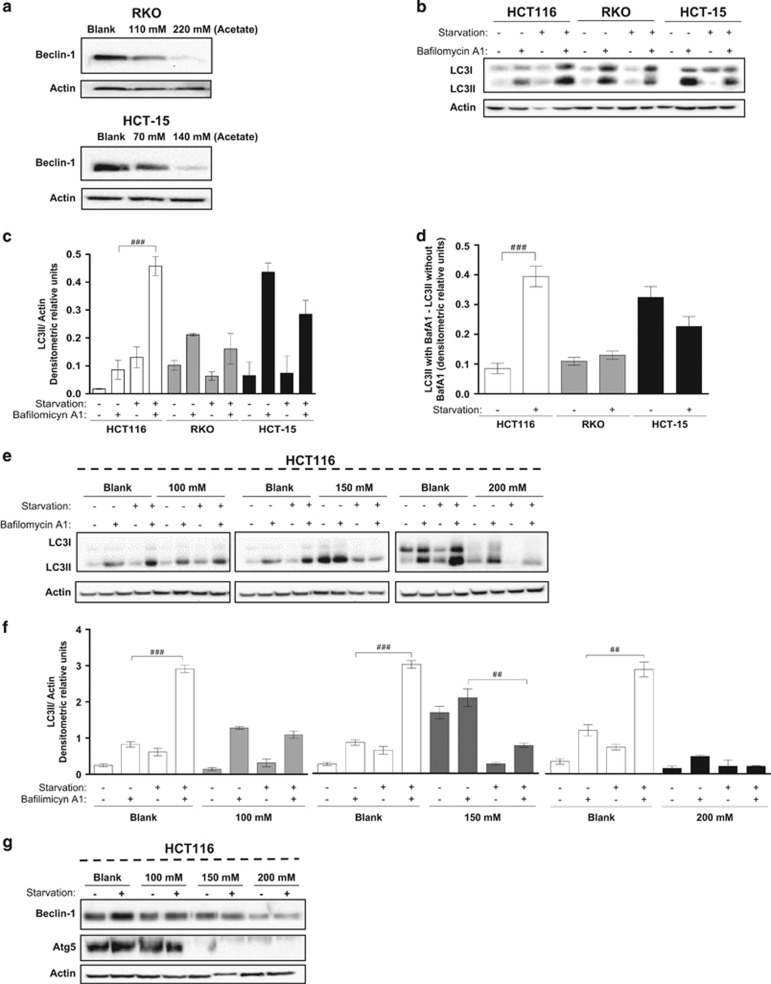
Acetate impairs autophagy in CRC cells. (**a**) Levels of Beclin-1 in cells exposed to acetate for 48 h (110 and 220 mM; 70 and 140 mM for RKO and HCT-15 cells, respectively) (**b**) Immunoblot analysis of LC3-I/II in HCT116, RKO and HCT-15 cell lines. Cells were maintained in complete medium or incubated in HBSS medium (starvation) for 6 h in the presence and absence of 20 nM bafilomycin A1 (Baf. A1). The HCT116 cell line exhibited the strongest autophagic response to nutrient limitation (starvation) and thus was chosen to address the effect of acetate on starvation-induced autophagy. (**c**) LC3-II/actin ratios of HCT116, RKO and HCT-15 cells were determined using Quantity One software (Bio-Rad manufacturer). (**d**) The autophagic flux was determined by subtracting the normalized LC3-II levels in the presence of Baf. A1 from the corresponding levels obtained in the absence of Baf. A1. (**e**) Levels of LC3-I/II and (**g**) of Beclin-1 and Atg5 in HCT116 cells exposed to acetate. Cells were left untreated (Blank) or treated with 100, 150 or 200 mM acetate for 42 h. Later, cells were maintained in complete medium or HBSS medium (starvation) with acetate for another 6 h in the presence or absence of 20 nM Baf. A1. These data demonstrate that acetate decreases the autophagic flux in CRC cells in a dose-dependent manner, as shown by the decrease in LC3-II delivery to the lysosome, and in Beclin-1 and Atg5 protein levels. (**f**) The LC3-II/actin ratios of HCT116 cells were determined using Quantity One software (Bio-Rad manufacturer). Actin was used as a loading control. Values are mean±S.E.M. of three independent experiments. Representative immunoblots are shown in (**a**, **b**, **e** and **g**). One-way ANOVA followed by Bonferroni's test, ^##^*P*≤0.01 and ^###^*P*≤0.001 compared with control cells treated with HBSS (starvation medium)

To determine if the increased mitochondrial mass resulting from acetate exposure could be due to autophagy inhibition, we monitored how it affects the levels of Beclin-1, a well-known autophagic player that is part of the class III PI3K complex. Acetate lowered Beclin-1 levels in both RKO and HCT-15 cells, consistent with inhibition of autophagy and possibly explaining the increased levels of mitochondrial proteins in acetate-treated cells (Figure 2a). We next determined whether acetate affects autophagy induction. We monitored starvation-induced autophagy (in HBSS medium) by assessing an autophagy hallmark: conversion of LC3-I to LC3-II^20^ (in the presence and absence of bafilomycin A1 (Baf. A1), a specific inhibitor of vacuolar type H^+^-ATPase used to inhibit the fusion of autophagosomes with lysosomes).^21^ HCT-15 and RKO cell lines had high basal autophagy levels and did not induce autophagy in response to starvation, evidenced by the increased accumulation of LC3-II in the presence of Baf. A1 already in control cells, which was not altered in response to starvation (Figures 2b–d). Therefore, we could not determine whether acetate affects autophagy induction in these cells. For that purpose, we instead used the HCT116 cell line, which displays increased autophagic flux in response to starvation (Figures 2b–d). We found that a 48 h exposure of HCT116 cells to IC_50_, intermediate concentration and 2 × IC_50_ of acetate (Supplementary Figure S4) decreased conversion of LC3-I to LC3-II in the presence of Baf. A1, which was even more pronounced under starvation conditions (Figures 2e and f). Increasing doses of acetate also progressively decreased the levels of Beclin1 and Atg5 proteins, essential for autophagosome completion,^22, 23^ in both complete and starvation media (Figure 2g). Taken together, our results indicate not only that exposure to acetate does not induce autophagy under our experimental conditions but also that it can inhibit both basal autophagy and autophagy induction.

### Active CatD mediates mitochondrial degradation and protects CRC cells from acetate-induced mitochondrial dysfunction

The cellular event or signal that determines whether mitochondrial degradation in cancer cells undergoing apoptosis occurs through selective or nonselective autophagy or by an autophagy-independent process, and which are the consequences to cell fate, is still unclear. In a previous study in *S. cerevisiae*, we showed that the yeast CatD (Pep4p) has an antiapoptotic role in acetic acid-induced cell death that depends on its proteolytic activity, and is required for mitochondrial degradation independently of autophagy.^14^ We therefore hypothesized that CatD, similarly to Pep4p, is required for efficient degradation of damaged mitochondria. To test this hypothesis, we assessed how CatD affects mitochondrial dysfunctions induced by acetate in CRC cells.

**Figure 3:**
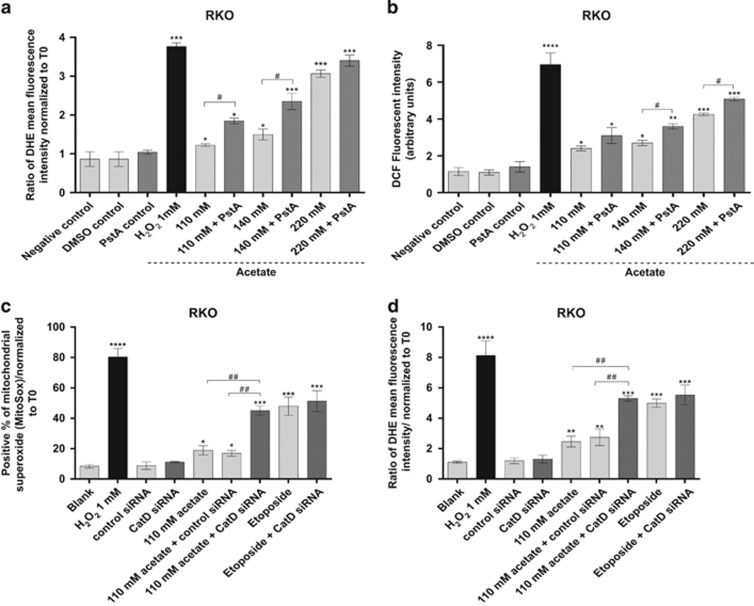
CatD protects CRC cells from oxidative stress. (**a** and **b**) Effect of PstA, a specific inhibitor of CatD catalytic activity, on ROS levels of RKO cells treated with acetate. PstA (100 *μ*M) was preincubated for 16 h and then coincubated with acetate for 24 h. (**a**) Accumulation of superoxide anion (O_2_^−^) in RKO cells was detected with dihydroethidium (DHE) and quantified by flow cytometry. (**b**) Accumulation of H_2_O_2_ in RKO cells was detected with H_2_DCF-DA using a fluorescence microplate reader. (**c** and **d**) Effect of CatD silencing on mitochondrial superoxide and total superoxide production in RKO cells treated with acetate (110 mM) or etoposide (50 *μ*M) for 48 h. As controls, RKO cells were not transfected (blank), transfected with scrambled control siRNA or transfected with CatD siRNA. H_2_O_2_ (1 mM) was used as a positive control for ROS production. (**c**) Accumulation of mitochondrial superoxide in RKO cells was detected with MitoSOX and quantified by flow cytometry. (**c**) Downregulation of CatD induces a significant increase in mitochondrial superoxide production normalized to time 0 (*T*0) in RKO cells treated with 110 mM acetate and (**d**) a significant accumulation of total superoxide, normalized to *T*0 cells. Values represent mean±S.E.M. of at least three independent experiments. *P≤0.05, **P≤0.01, ***P≤0.001 and ****P≤0.0001 compared with control cells. ^#^*P*≤0.05 and ^##^*P*≤0.001 comparing acetate treatment with acetate/PstA or acetate treatment with acetate/CatD siRNA

We first analyzed whether Pepstatin A (PstA), a known specific pharmacological inhibitor of CatD, or transfection with specific RNA interference (siRNA) targeting CatD interfered with acetate-induced accumulation of ROS in RKO cells. We found that PstA increased total O_2_^−^ and H_2_O_2_ accumulation in cells exposed to the three acetate concentrations for 24 h (Figures 3a and b). Downregulation of CatD was even more effective, as it significantly increased total and mitochondrial O_2_^−^ accumulation in cells treated with 110 mM acetate (detected with DHE or MitoSOX, respectively) (Figures 3c and d). In contrast, inhibition of CatD did not significantly affect etoposide-induced ROS accumulation (Figures 3c and d).

**Figure 4:**
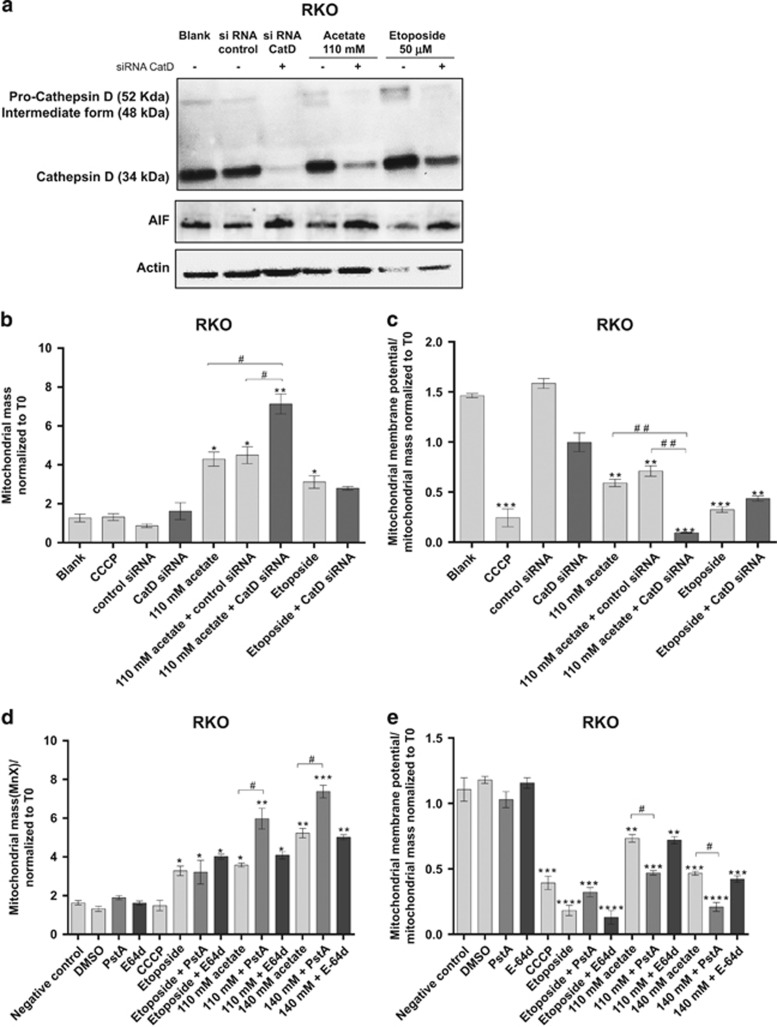
CatD is involved in autophagy-independent mitochondrial degradation in CRC cells. (**a**) Western blot of CatD isoforms (Pro-CatD, intermediate form and mature CatD) and AIF with or without CatD silencing in the absence or presence of acetate (110 mM) or etoposide (50 *μ*M). As controls, RKO cells were not transfected (blank), transfected with scrambled control siRNA or transfected with CatD siRNA. Actin was used as a loading control. (**b** and **c**) Effect of CatD silencing on mitochondrial dysfunction in RKO cells treated with acetate (110 mM) or etoposide (50 *μ*M) for 48 h. (**b**) Downregulation of CatD induces a significant increase in mitochondrial mass normalized to time 0 (*T*0) in RKO cells treated with 110 mM acetate and (**c**) a significant mitochondrial membrane depolarization normalized to mitochondrial mass, in relation to *T*0. For each bar, the mean value for at least three independent experiments is represented. Bonferroni's test, *P≤0.05; **P≤0.01 and ***P≤0.001 compared with negative control cells. ^#^*P*≤0.05, ^##^*P*≤0.01 comparing acetate treatment with acetate/CatD siRNA. (**d** and **e**) Effect of the specific CatD inhibitor (PstA) and the CatB and CatL inhibitor (E-64d) on mitochondrial dysfunction in RKO cells treated with acetate (110 and 140 mM) or etoposide (50 *μ*M) for 48 h. E-64d (10 *μ*M) and PstA (100 *μ*M) were pre-icubated for 1 or 16 h, respectively, and then coincubated with acetate or etoposide for 48 h. Cells were incubated with fresh complete medium or with 50 *μ*M CCCP as a negative and positive control, respectively. Cells were then double stained with 400 nM MitoTracker Green and 200 nM MitoTracker Red CMXRos for 30 min at 37 ºC in the dark and analyzed by flow cytometry. Inhibition of CatD activity with PstA induces an increase in mitochondrial mass (**d**) and membrane depolarization normalized to mitochondrial mass in relation to *T*0 in RKO cells (**e**). For each bar, the mean value for at least three independent experiments is represented. Bonferroni's test *P≤0.05; **P≤0.01, ***P≤0.001 and ****P≤0.0001 compared with negative control cells. ^#^*P*≤0.05 comparing acetate treatment with acetate/PstA

We next tested the effect of depleting CatD on mitochondrial mass. Transfection with CatD siRNA clearly increased the levels of the mitochondrial protein AIF in both acetate- and etoposide-treated RKO cells, although not in untreated controls (Figure 4a). Using the double-staining protocol described above, we also found that depletion of CatD in acetate-treated RKO cells significantly increased mitochondrial mass and decreased ΔΨm. In contrast, it had no significant effect in cells treated with etoposide (Figures 4b and c). Altogether, these data indicate that CatD protects RKO cells from mitochondrial dysfunction induced by acetate through its involvement in the degradation of damaged mitochondria.

**Figure 5:**
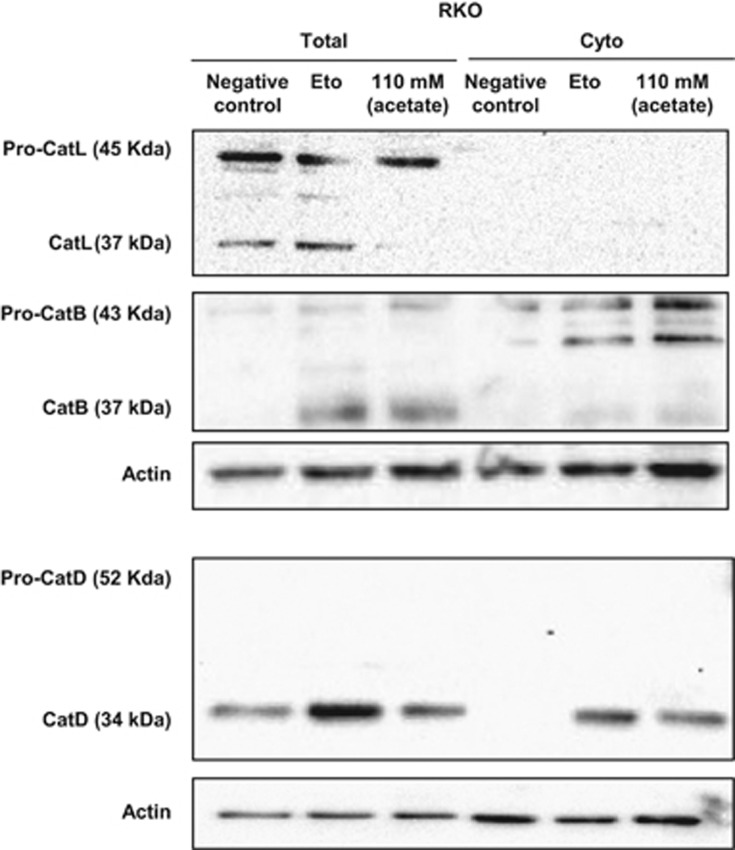
Acetate induces specific CatD release to the cytosol in CRC cells. Effect of acetate on the expression and release of CatL, CatB and CatD to the cytosol in RKO cells. Cells were treated with 110 mM acetate or 50 *μ*M etoposide for 48 h, or with fresh medium as a negative control. Total and cytosolic fractions (Cyto) are shown. Actin was used as a loading control

To further analyze whether the role of CatD in mitochondrial degradation depends on its proteolytic activity, we assessed how this parameter was affected by inhibition with PstA. PstA significantly increased the mitochondrial mass of RKO cells exposed to 110 and 140 mM acetate for 48 h, but decreased the ΔΨm (Figures 4d and e). In contrast, inhibition of CatD in RKO cells exposed to etoposide did not affect mitochondrial mass or the ΔΨm. Moreover, E-64d, an inhibitor of CatB and CatL (also overexpressed in CRC cells),^24^ had no effect on the mitochondrial mass or ΔΨm of either acetate- or etoposide-treated cells (Figures 4d and e). We confirmed that E-64d was active under our experimental conditions because it decreased caspase-3 activity in cells exposed to etoposide (not shown), confirming the previously described proapoptotic function of CatB and CatL in this process.^25^ These results indicate that CatD, but not CatL or CatB, is involved in mitochondrial degradation in response to acetate. Accordingly, we found that only the active form of CatD, but not CatL and only trace amounts of CatB, is released to the cytosol after acetate treatment (Figure 5).

**Figure 6:**
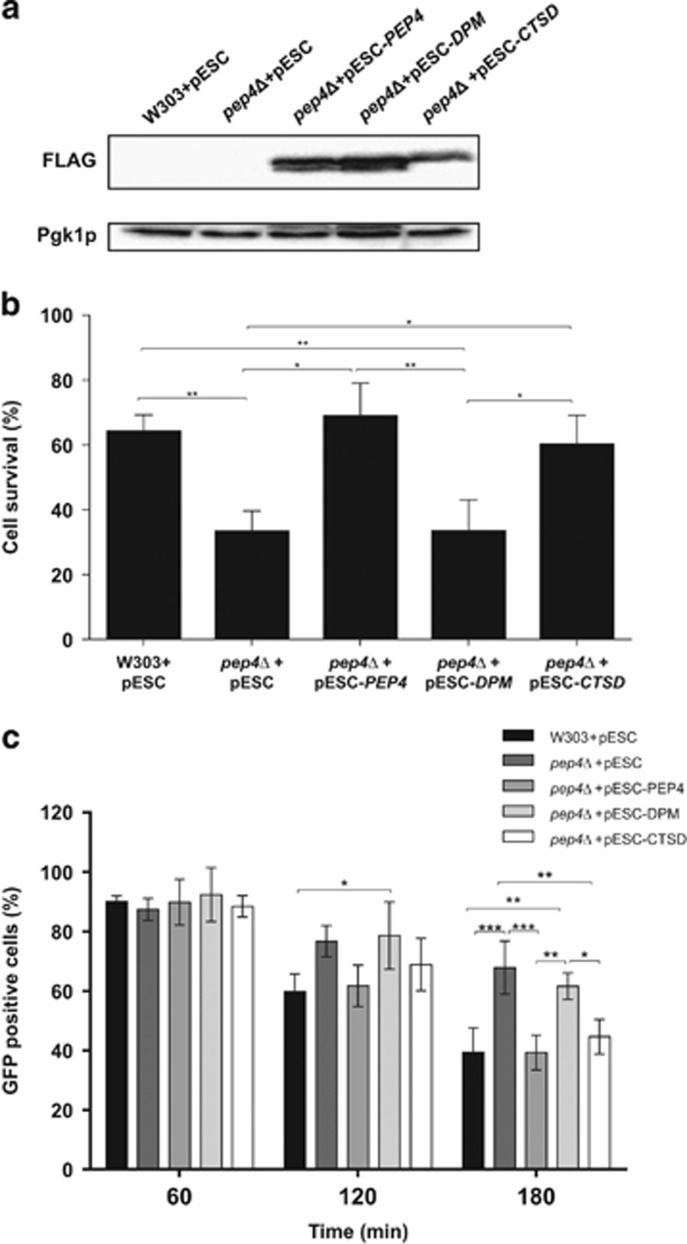
Cell survival and mitochondrial degradation in *S. cerevisiae* cells during acetic acid treatment. The W303 strain transformed with the empty vector (pESC) and *pep4*Δ strains, transformed with the empty vector (pESC), pESC-*PEP4* (expressing WT-Pep4p), pESC-*DPM* (expressing DPM-Pep4p) or pESC-*CTSD* (expressing human CatD), were incubated with 120 mM acetic acid for up to 180 min. (**a**) Immunoblot analysis of whole-cell extracts of *pep4*Δ cells expressing FLAG-tagged WT-Pep4p, FLAG-tagged DPM-Pep4p, FLAG-tagged CatD and the corresponding empty vector after 20 h of growth. Pgk1p was used as a loading control. (**b**) Cell survival at time 180 min was determined by standard dilution plate counts and expressed as a percentage of colony-forming units (CFU) in relation to time 0. Data represents means±S.D. (*n=3*). (**c**) Mitochondrial degradation was assessed by measuring the percentage of cells without loss of mtGFP fluorescence (100% corresponds to the number of GFP positive cells at time 0). Data represent means±S.D. (*n=4*). *P<0.05, **P<0.01 and ***P<0.001

The aforementioned results indicate that the role of CatD in mitochondrial degradation depends on its proteolytic activity. To further support this conclusion and determine whether this mechanism is conserved, we assessed if the same is true for the yeast CatD, and if human and yeast CatD are functionally equivalent. For this purpose, we constructed strains deficient in Pep4p expressing the empty vector control, and equivalent levels of FLAG-tagged wild-type Pep4p, a double-point mutant form deficient in proteolytic activity, and human CatD (Figure 6a). We then compared their sensitivity to acetic acid with that of wild-type W303 cells expressing the empty vector. In accordance with our previous data,^14^ expression of wild-type Pep4p, but not of the catalytically inactive mutant, reverted the sensitivity of Pep4p-deficient cells to acetic acid-induced apoptosis. Now, we further show that expression of human CatD also compensates for the loss of Pep4p, indicating that the two proteins have a similar role in this process (Figure 6b). All strains were then transformed with a plasmid expressing mitochondrial GFP, and mitochondrial degradation in response to acetic acid was assessed by estimating the percentage of cells with preserved green fluorescence, as described previously.^14^ While expression of wild-type Pep4p and CatD reverted the delay in mitochondrial degradation of Pep4p-deficient cells exposed to acetic acid, expression of double-point mutant Pep4p did not (Figure 6c). Although the precise mechanism underlying the role of Pep4p in cell survival and in mitochondrial degradation is still elusive, we have now determined that both depend on its proteolytic activity and are complemented by CatD. Taken together, our results show that Pep4p/CatD is involved in acetic acid/acetate-induced mitochondrial degradation independently of autophagy but dependently of its catalytic activity in both yeast and CRC cells.

## Discussion

CRC is one of the most common solid tumors worldwide.^2, 26^ A diet rich in dietary fiber is associated with a reduction in CRC incidence,^1, 27^ indicating CRC may be amenable to prevention through a dietary regimen.^15, 16, 27^ Some of the significant health benefits of dietary fiber can be attributed to its microbial fermentation, namely by Propionibacteria in the colon into SCFAs (acetate, propionate and butyrate).^1, 6, 15, 16, 28^ Indeed, many studies suggest these SCFAs protect against carcinogenesis, as they reduce human colon cancer cell growth and differentiation and stimulate apoptosis in CRC cells.^4, 6, 27, 29^ Acetate is one of the most important SCFAs, but has been less investigated than propionate and butyrate. Nonetheless, previous studies proposed that acetate inhibits proliferation and induces apoptosis in colon cancer cells^3, 4, 5, 6^ and that acetate-induced apoptosis in CRC cells involves different biochemical events, including mitochondrial alterations.^3, 4, 5^ We have also previously shown that acetate induces apoptosis in CRC-derived cells in a dose-dependent manner, characterized by DNA fragmentation, caspase-3 activation, phosphatidylserine exposure to the outer leaflet of the plasma membrane and the appearance of a sub-G1 population^5^. In addition, we showed that this process is associated with lysosomal alkalinization, LMP and CatD release into the cytosol.^5^

It is well established that partial LMP followed by a release of lysosomal hydrolases into the cytosol can activate intrinsic caspase-dependent apoptosis or a caspase-independent alternative cell death pathway.^7, 8^ Of these, CatD has emerged as an important molecular target in cancer therapy, as it is overexpressed and secreted by cells of various tumor types, including CRC.^30, 31^ CatD has a vital role in extracellular matrix degradation and cancer cell survival, and actively participates in the invasion of carcinoma cells during both local invasion and metastasis formation.^32, 33^ However, its role in apoptosis depends on cellular types, specific contexts, stimulus and catalytic activity.^10, 33^ Our data indicate that CatD has a protective role in acetate-induced apoptosis in CRC cells,^5^ as we had shown for its ortholog Pep4p in *Saccharomyces cerevisiae.*^13^ As SCFAs, and in particular acetate, have garnered increased interest as potential prevention/therapeutic agents in CRC, we sought to further understand the mechanism underlying the antiapoptotic role of this lysosomal protease under this specific cellular and stimulus context. Based on data from our group and others, as well as on the present study, we propose that the lysosome and mitochondria communicate during acetate-induced apoptosis in CRC cells through permeabilization of both organelles and selective leakage of proteins. Indeed, we show that acetate triggers release of CatD to the cytosol, but not of CatL, and only a small amount of CatB, which does not seem to have major consequences. Besides lysosomal alterations, acetate triggered mitochondrial dysfunction in a dose- and time-dependent-manner, characterized by increased mitochondrial ROS, changes in ΔΨ*m* and an increase in mitochondrial mass, which were enhanced when CatD was inhibited. This mitochondrial dysfunction is in agreement with that reported during apoptosis induction by acetate and a mixture of acetate and propionate produced by *Propionibacterium freudenreichii* in other CRC cells (HT-29), including increased ROS and ΔΨm dissipation, as well as swelling in isolated mitochondria.^4^ However, the role of CatD or the lysosome in that context was not evaluated.

The increase in mitochondrial mass observed after exposure of cells to acetate led us to investigate whether the presumed decrease in mitochondrial turnover was associated with modulation of autophagy by this SCFA. Previous studies demonstrated that apoptosis triggered by low doses (1–10 mM) of propionate and butyrate can be delayed because autophagy is also induced, which can potentially impair the therapeutic efficacy of SCFAs in colon cancer.^15, 16, 17^ Indeed, autophagy has been implicated in cancer progression, used by cells for autophagic degradation of damaged organelles, long-lived proteins and pathogens, and in this way maintain homeostasis.^20, 29, 34^ Further assays are required to ascertain whether low concentrations of acetate can cause a similar adaptive response in CRC cells. However, we previously showed that autophagy is not induced during acetic acid-induced apoptosis in yeast.^13^ In the present study, we demonstrate that acetate impairs the autophagic flux of HCT116 cells and decreases Beclin-1 levels in all three cell lines. Therefore, we show that concentrations of acetate normally present in the human intestine have a previously uncharacterized effect in CRC cells: inhibition of autophagy, most likely through impairment of autophagosome and lysosome fusion during acetate-induced apoptosis.

It is known that malfunctioning mitochondria are selectively targeted for autophagic degradation.^16^ However, it is conceivable that cells use alternative pathways to clear damaged mitochondria when autophagy is inactive. We therefore investigated if CatD is involved in mitochondrial degradation, similar to Pep4p in yeast, possibly having a prosurvival role through elimination of dysfunctional mitochondria. We show that CatD protects RKO cells from acetate-induced oxidative stress and mitochondrial depolarization, as its inhibition with PstA or downregulation with siRNA cause a significant increase in the levels of H_2_O_2_ and O_2_^−^ and a decrease in ΔΨm. Hah *et al.*^10^ have previously suggested that CatD has a protective role in H_2_O_2_-induced cell death in M059J glioblastoma cells through its involvement in autophagy, which enhances cell survival under oxidative stress.^10^ Accordingly, we had shown that Pep4p deletion increased the sensitivity of yeast cells to acetic acid associated with increased ROS accumulation,^14^ although autophagy was not induced.^13^ These data, together with our results, support the notion that reduced CatD activity leads to increased ROS accumulation and mitochondrial depolarization. We therefore proceeded to determine whether CatD is involved in the degradation of damaged mitochondria, as we presumed to be the case during acetate-induced apoptosis in CRC cells. We found that downregulation of CatD in acetate-treated RKO cells increased mitochondrial mass, as well as the cellular pool of AIF. These findings corroborate our hypothesis that CatD is involved in the degradation of damaged mitochondria during acetate-induced apoptosis through an autophagy-independent process. Although not sufficiently efficient to maintain mitochondrial mass homeostasis, as an acetate-induced increase in mitochondrial mass is still observed, this alternative degradation process allows the cell to dispose of dysfunctional mitochondria and delay cell death. It will be interesting in the future to determine if CatD is also released and is involved in mitochondrial degradation in response to other SCFAs, either directly or through downstream substrates. Inhibition of CatD with PstA mimicked the results obtained with CatD siRNA, indicating the catalytic activity of CatD is required for this function, as we previously found for the antiapoptotic role of both yeast and human CatD.^5, 14^ We now also show that the role of yeast CatD in acetic acid-induced mitochondrial degradation depends on its proteolytic activity and can be complemented by human CatD, indicating this mechanism is conserved through evolution.

As discussed above, partial LMP followed by a release of lysosomal hydrolases into the cytosol can activate intrinsic caspase-dependent apoptosis or a caspase-independent alternative cell death pathway.^7, 8^ Thus, disabling lysosome function is under investigation as an adjuvant therapeutic approach to sensitize cells to apoptosis-inducing agents.^7^ Destabilization of the lysosomal membrane has been proposed as a promising strategy, as it would promote apoptosis through initiation of the lysosomal pathway in cancer cells.^8^ However, our results show that the release of lysosomal proteases, in particular of CatD,^5^ may enable a degradation process alternative to autophagy with a similar protective role in cell survival, thus counteracting activation of the lysosomal death pathway. This would negatively impact the efficacy of acetate, and probably of other therapeutic compounds that trigger LMP and impair autophagy, and would require inhibiting CatD in combination treatments.

In summary, our findings based on the clues provided by the yeast system unveiled a novel prosurvival function of CatD in autophagy-independent mitochondrial degradation, which can enhance cell survival in CRC cells undergoing acetate-induced apoptosis. These results offer new perspectives in the intricate regulation of life and death, and suggest using CatD inhibitors as adjunct therapy with SCFAs for prevention/therapy of CRC.

## Materials and Methods

### Cell lines and culture

We used three cell lines derived from human CRC, namely RKO^*BRAFV600E*^, HCT-15^*KRASG13D*^ and HCT116^*KRASG13D; PI3KCA*^. Cells were maintained at 37 °C under a humidified atmosphere containing 5% CO_2_. HCT-15 cells were grown in RPMI (Biowest, Nuaillé, France) medium with l-glutamine and HEPES. RKO cells were grown in DMEM (Biowest) with high glucose and supplemented with 1 mM sodium pyruvate and 1.5 g/l sodium bicarbonate. HCT116 cells were grown in McCoy's 5 A (Biowest) medium. All culture media were supplemented with 10% fetal bovine serum and 100 U/ml penicillin/streptomycin. For autophagy studies, we used HBSS (Gibco, Paisley, UK) medium supplemented with 7.5% sodium bicarbonate.

### Cell treatments with acetate

Cells were seeded and adhered onto appropriate sterile plates for 24 h before treatments in all experiments. We previously determined the IC_50_, IC intermediate (IC inter) and 2 × IC_50_ of acetate after 48 h of treatment: 70, 100 and 140 mM for HCT-15; 110, 140 and 220 mM for RKO cell lines, respectively.^5^ The IC_50_, IC inter and 2 × IC_50_ values for HCT116 (100, 150 and 200 mM, respectively) were calculated from the mean values of sulforhodamine B reduction as described in Marques *et al.*^5^ (Supplementary Figure S3). For each bar, the mean value of at least three independent experiments is represented. Bonferroni's test: *P≤0.05, **P≤0.01 and ***P≤0.001 compared with negative control cells.

### RNA interference-mediated inhibition of CatD

RKO cells were plated in 6-well plates at a density of 3 × 10^4^ cells per well. After 24 h, about 70% confluence was confirmed before transfection with small interfering RNA (siRNA) oligonucleotides. Cells were transfected with 100 nM on-target plus SMART pool siRNA against CatD (A-003649-16; Thermo Fisher Scientific, Lafayette, CO, USA). Transfection performance was monitored using a validated Silencer Select Negative Control (scrambled siRNA control, no. 4390843; Life Technologies, Carlsbad, CA, USA). Transfection was performed with 6 *μ*l Lipofectamine 2000 (Invitrogen Corp., Paisley, UK). After 14 h, the transfection mixture was removed and cells were left untreated (blank) or treated with acetate (110 mM) or etoposide (50 *μ*M) and incubated for a further 48 h in fresh medium. MitoSOx, DHE and double staining with MitoTracker Green and Mitotracker Red CMXRos were performed as described below. The experiments were carried out in three replicates, and CatD levels were monitored by western blotting.

### Determination of intracellular ROS

#### Dichlorofluorescein assay

Cells were seeded in a 12-well plate at a density of 1.4 *×* 10^5^ and 1.8 *×* 10^5^ cells per well (RKO and HCT-15 cells, respectively). When indicated, cells were preincubated with PstA (Sigma-Aldrich, St Louis, MO, USA) for 16 h and then coincubated with PstA without and with different acetate concentrations for 12, 24 and 48 h. After treatment, cells were washed two times with 1x PBS and H_2_DCF-DA (Sigma-Aldrich) was added to the culture plates at a final concentration of 100 *μ*M. Plates were incubated at 37 °C for 30 min in the dark. Then, cells were lysed with 500 *μ*l of 90% DMSO/10% PBS (lysis solution) for 10 min at room temperature in the dark, with agitation. H_2_O_2_ (500 *μ*M or 1 mM for HCT-15 or RKO cell lines, respectively) was used as a positive control. H_2_DCF-DA fluorescence intensity was detected with emission wavelength at 538 nm and excitation wavelength at 485 nm using a fluorescence microplate reader (Fluoroskan Ascent FL; Thermo Fisher Scientific Inc., Waltham, MA, USA). Values are expressed as the mean fluorescence intensity normalized to the cell number in each condition at the end of the experiment. Three independent experiments were performed.

#### DHE assay

Non-treated cells (negative control) or cells exposed to different acetate concentrations for 12, 24 and 48 h were analyzed. When used, cells were preincubated with PstA for 16 h and then coincubated with PstA without and with different acetate concentrations for 24 h. Inhibition of CatD in RKO cells was performed as described above. Approximately 1 × 10^6^ floating and attached cells were collected, washed with 1 × PBS, centrifuged at 1500 × *g* for 5 min and incubated with 150 nM DHE (Molecular Probes, Eugene, OR, USA) (30 min, 37 °C, in the dark) to detect superoxide anion (O_2_^−^). Fluorescence emission of oxidized DHE was analyzed by flow cytometry. H_2_O_2_ (500 *μ*M or 1 mM for HCT-15 or RKO cell lines, respectively) was used as a positive control. Values are expressed as the mean of fluorescence intensity normalized to *T*0 (control for ROS level before the treatment). Three independent experiments were carried out and data were analyzed using the Flowing software (version 2.5.1, Turku Centre for Biotechnology,Turku, Finland).

#### MitoSOX assay

MitoSOX Red (Molecular Probes) was used to detect mitochondrial superoxide. Approximately 1 × 10^6^ floating and attached cells were collected, washed with 1x PBS, centrifuged at 1500 × *g* for 5 min and incubated with 2.5* μ*M MitoSOX Red for 30 min at room temperature. Fluorescence emission of oxidized MitoSOX Red was analyzed by flow cytometry. H_2_O_2_ (1 mM) was used as a positive control. Values are expressed as the percentage of cells with positive staining normalized to *T*0 (control for ROS level before the treatment).

### Analysis of mitochondrial alterations

#### Mitochondrial mass and ΔΨm analysis

MitoTracker Green FM (Molecular Probes) was used to analyze the relative mitochondrial mass. MitoTracker Green is a green fluorescent dye that localizes to the mitochondrial matrix regardless of the ΔΨm and covalently binds to mitochondrial proteins by reacting with free thiol groups of cysteine residues. MitoTracker Red CMXRos (Molecular Probes) was used simultaneously, to monitor the changes in ΔΨm. MitoTracker Red CMXRos is a red-fluorescent dye that stains mitochondria in live cells through accumulation in mitochondria in a membrane potential-dependent manner. Untreated cells (negative control), cells exposed to etoposide (50 *μ*M), an inducer of LMP, or to different acetate concentrations were analyzed at different time points. When used, cells were preincubated with 10 *μ*M (2*S*,3*S*)-*trans*-epoxysuccinyl-l-leucylamido-3-methylbutane ethyl ester (E-64d; Sigma-Aldrich) (a CatB and CatL inhibitor) or 100 *μ*M PstA for 1 and 16 h, respectively, and then co-incubated with different acetate concentrations or etoposide for 48 h. Both floating cells and attached cells were collected, washed with 1x PBS, centrifuged at 1500 × *g* for 5 min and incubated with 400 nM MitoTracker Green FM and 200 nM MitoTracker Red CMXRos (30 min, 37 °C, in the dark). The uncoupling agent CCCP (50 *μ*M, 30 min) was used as positive control for mitochondrial membrane depolarization. Fluorescence emission was analyzed by flow cytometry. The values of mitochondrial mass for each time point were expressed as the ratio between the mean green fluorescence intensity and the one correspondent to time zero (*T*0). The values of the ΔΨm for each time point were expressed as the ratio between the mean red fluorescence intensity and the mean green fluorescence intensity normalized to the correspondent one at *T*0. Three independent experiments were carried out.

#### Fluorescence microscopy studies

Cells were seeded in 6-well plates containing glass coverslips and exposed to acetate for 48 h. At the end of the experiment, cells were washed with 1x PBS and incubated with 20 nM DiOC_6_(3) (Molecular Probes) or with 400 nM MitoTracker Green and 200 nM MitoTracker Red CMXRos for 30 min, at 37 °C, in the dark. CCCP (50 *μ*M, 30 min) was also used as a positive control for ΔΨm loss. Coverslips were mounted in 1x PBS and immediately observed under the fluorescence microscope (Leica DM 5000B; Leica Microsystems, Wetzlar, Germany). Three coverslips were prepared for each experimental condition and representative images are shown.

#### Flow cytometry analysis

Samples were analyzed in a flow cytometer (Epics XL; Beckman Coulter, Miami, FL, USA) equipped with an argon-ion laser emitting a 488-nm beam at 15 mW. Detection of red fluorescence was performed using FL-4 (488/620 nm) and detection of green fluorescence was performed using FL-1 (488/525 nm). A total of 30 000 cells were acquired *per* sample and data were analyzed using the Flowing software (version 2.5.1, Turku Centre for Biotechnology).

#### Assessing autophagy in CRC cell lines

Cell lines untreated (Blank) or treated with IC_50_, IC inter or 2 × IC_50_ acetate concentrations were incubated for 42 h in complete medium. Later, cells were incubated in HBSS medium for 6 h in the presence or absence of 20 nM Baf. A1 (Acros Organics, Geel, Belgium), in the absence or presence of acetate.

#### Cell survival and mitochondrial degradation assays in yeast

*Saccharomyces cerevisiae* strain W303-1A was used as the wild-type strain. The *pep4*Δ mutant was constructed in W303-1A by homologous recombination using a *PEP4*::*kanMX4* disruption cassette amplified from the respective Euroscarf deletion strain by PCR. Correct integration of the cassette was confirmed by PCR. To construct CatD^FLAG^, the insert was amplified by PCR from the plasmid pJP1520-*CTSD* (containing human CatD cDNA) and integrated by homologous recombination into the pESC-His vector. Correct integration was verified by sequencing. The W303-1A strain was transformed with the empty vector (pESC) and the *pep4*Δ strain was transformed with the empty vector (pESC), pESC-*PEP4*, pESC-*DPM* and pESC-*CTSD* plasmids for expression of WT-Pep4, double-point mutant (DPM-Pep4p) or human CatD, respectively. The strains were transformed with the pGAL-CLbGFP (see Okamoto *et al.*^35^) vector for mitochondrial degradation studies. Strain growth conditions, acetic acid treatments and mitochondrial degradation assays were performed as described previously.^14^

#### Preparation of total/cytosolic protein extracts and western blotting

Cell lysis of mammalian cells, protein sample preparation (total and cytosolic) and western blotting were carried out as described previously^5^ with 25 *μ*g of total or cytosolic extracted proteins applied per lane before SDS-PAGE. Total yeast extracts were prepared by suspending ~2 × 10^6^ cells in 0.5 ml of water and adding 50 *μ*l of a mixture of 3.5% *β*-mercaptoethanol in 2 M NaOH. After a 15 min incubation on ice, proteins were precipitated with 50 *μ*l of 3 M trichloracetic acid for 15 min on ice. After a rapid centrifugation, the pellet was resuspended in Laemmli buffer for SDS-PAGE.

The primary antibodies used were anti-Beclin-1 (1 : 3000; Cell Signaling Technology, Leiden, Netherlands), anti-LC3-II (1 : 5000; Sigma-Aldrich), anti-Atg5 (1 : 3000; Sigma-Aldrich), anti-CatD (1 : 100; Calbiochem, San Diego, CA, USA), anti-CatB (1 : 300; Abcam, Cambridge, UK), anti-CatL (1 : 1000; Abcam), anti-AIF (internal) rabbit polyclonal antibody (1 : 500; Chemicon International, Billerica, MA, USA ), anti-VDAC1 (1 : 2000; MitoSciences, Eugene, OR, USA), anti-Tom22 (FL-145) (1 : 1000; Santa Cruz Biotechnology, Santa Cruz, CA, USA), anti-actin (Santa Cruz Biotechnology, Santa Cruz), anti-FLAG (1 : 5000; Sigma-Aldrich) and anti-yeast phosphoglycerate kinase (PGK1) antibody (1 : 5000; Molecular Probes). Secondary antibodies used were peroxidase-conjugated AffiniPure goat anti-rabbit IgG (Jackson ImmunoResearch, West Grove, PA, USA) and horseradish peroxidase-labeled goat anti-mouse immunoglobulin IgG (Jackson ImmunoResearch). Subsequent chemiluminescence detection was performed using the ECL detection system (Amersham, Biosciences, Buckinghamshire, UK) and a molecular imager by Chemi-Doc XRS system (Bio-Rad, Laboratories Inc., Hercules, CA, USA). When performed, densitometry analysis of protein bands was performed using the Quantity One software (version 4.6.9, Bio-Rad manufacturer, Hercules, CA, USA) and levels of actin used as a normalization control for protein loading.

#### Statistical analysis

Data are expressed as mean±S.D. of three independent experiments. Statistical significance analysis was determined by one-way ANOVA followed by Dunnett or Bonferroni's test for multiple comparisons with the control using Prism software (GraphPad, La Jolla, CA, USA). The differences were considered significant for *P-*values lower than 0.05.

## Abbreviations

BafA1: bafilomycin A1
CatB: cathepsin B
CatD: cathepsin D
CatL: cathepsin L
CRC: colorectal carcinoma
CCCP: carbonyl cyanide m-chlorophenylhydrazone
E-64d: (2*S*,3*S*)-*trans*-epoxysuccinyl-l-leucylamido-3-methylbutane ethyl ester
ΔΨm: mitochondrial membrane potential
LMP: lysosomal membrane permeabilization
ROS: reactive oxygen species
PstA: pepstatin A
SCFA: short-chain fatty acids
SRB: sulforhodamine B
