# Supplementary material for: Cathepsin D protects colorectal cancer cells from acetate-induced apoptosis through autophagy-independent degradation of damaged mitochondria
Source: Cell Death Dis. 2015 Jun 18;6(6):e1788–. doi: 10.1038/cddis.2015.157 (PMC4669836; doi:10.1038/cddis.2015.157)
Supplement: Supplementary Figures [file cddis2015157x1.ppt]

## Slide 1
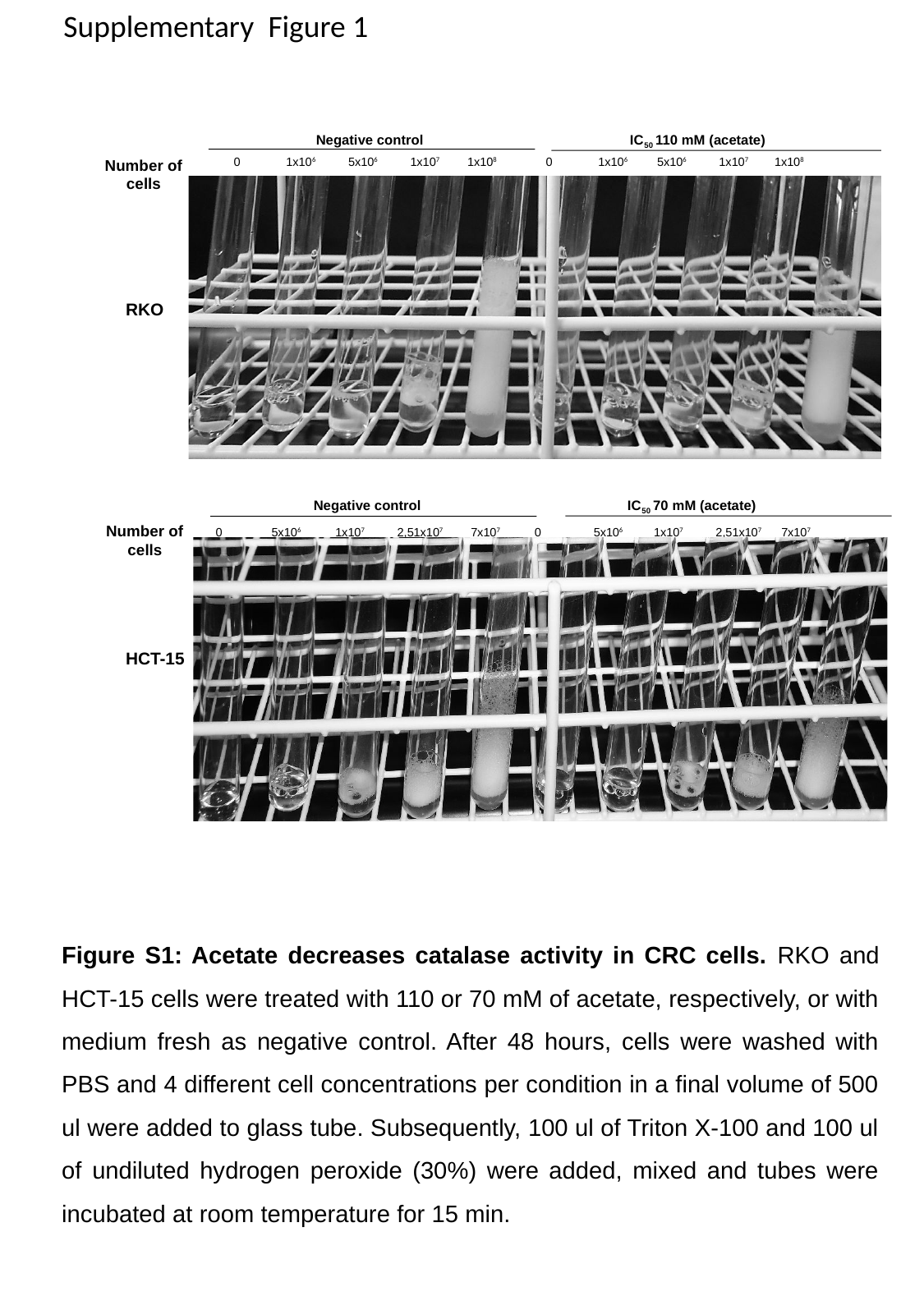

Supplementary Figure 1
Negative control IC50 110 mM (acetate)
0 1x10^6^ 5x10^6^ 1x10^7^ 1x10^8^ 0 1x10^6^ 5x10^6^ 1x10^7^ 1x10^8^
Number of
 cells
RKO
HCT-15
 Negative control IC50 70 mM (acetate)
 0 5x10^6^ 1x10^7^ 2,51x10^7^ 7x10^7^ 0 5x10^6^ 1x10^7^ 2,51x10^7^ 7x10^7^
Number of
 cells
Figure S1: Acetate decreases catalase activity in CRC cells. RKO and HCT-15 cells were treated with 110 or 70 mM of acetate, respectively, or with medium fresh as negative control. After 48 hours, cells were washed with PBS and 4 different cell concentrations per condition in a final volume of 500 ul were added to glass tube. Subsequently, 100 ul of Triton X-100 and 100 ul of undiluted hydrogen peroxide (30%) were added, mixed and tubes were incubated at room temperature for 15 min.

## Slide 2
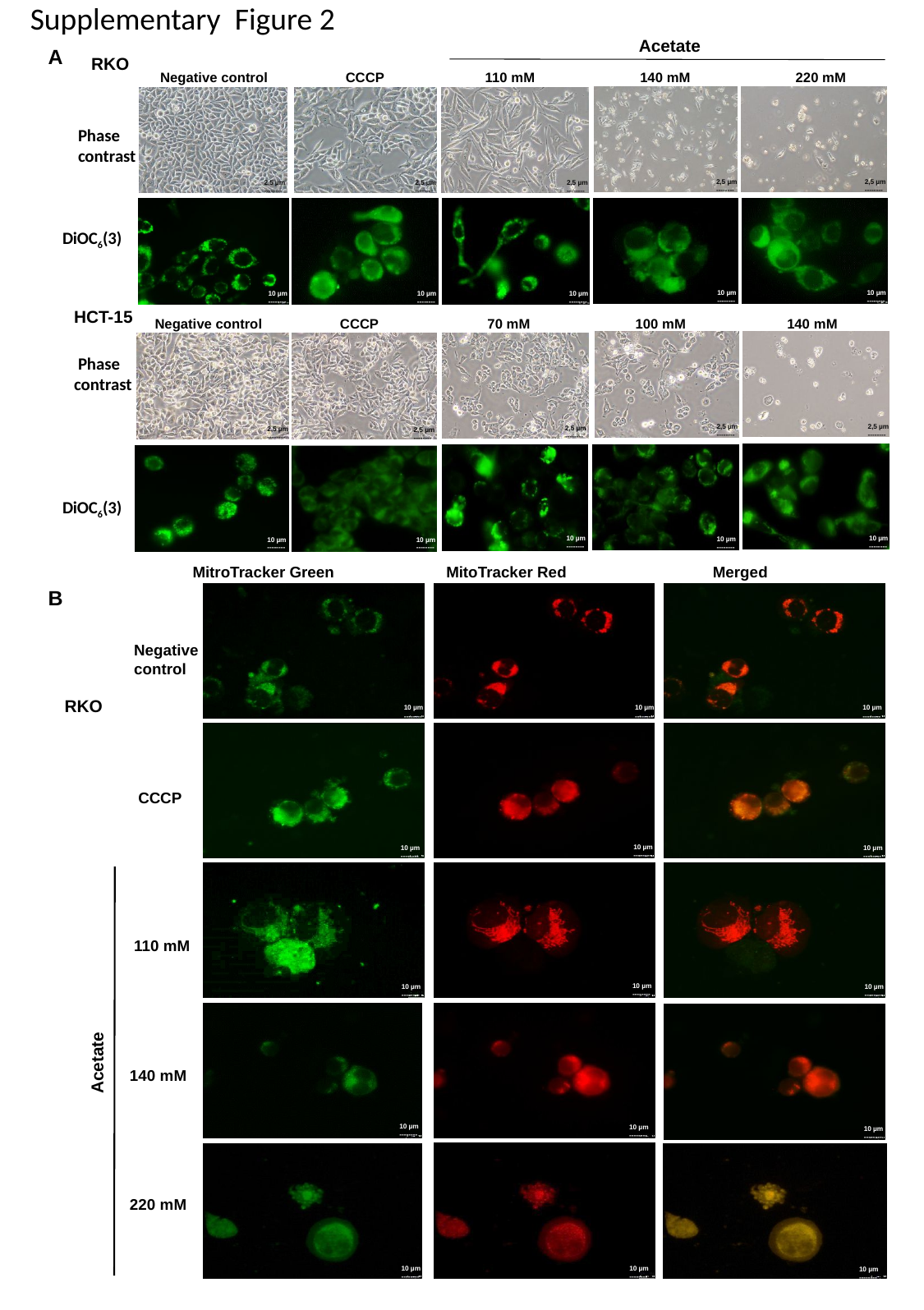

RKO
 HCT-15
RKO
Supplementary Figure 2
Acetate
A
B
 Negative control CCCP 110 mM 140 mM 220 mM
 Phase
 contrast
DiOC6(3)
 Phase
 contrast
DiOC6(3)
2,5 µm
--------
2,5 µm
--------
2,5 µm
--------
2,5 µm
--------
2,5 µm
--------
25 µm
--------
10 µm
--------
10 µm
--------
10 µm
--------
10 µm
--------
10 µm
--------
 Negative control CCCP 70 mM 100 mM 140 mM
2,5 µm
--------
2,5 µm
--------
2,5 µm
--------
2,5 µm
--------
2,5 µm
--------
25 µm
--------
10 µm
--------
10 µm
--------
10 µm
--------
10 µm
--------
10 µm
--------
MitroTracker Green MitoTracker Red Merged
 Negative
 control
 CCCP
 110 mM
 140 mM
 220 mM
10 µm
--------
10 µm
--------
10 µm
--------
10 µm
--------
10 µm
--------
10 µm
--------
10 µm
--------
10 µm
--------
10 µm
--------
Acetate
10 µm
--------
10 µm
--------
10 µm
--------
10 µm
--------
10 µm
--------
10 µm
--------

## Slide 3
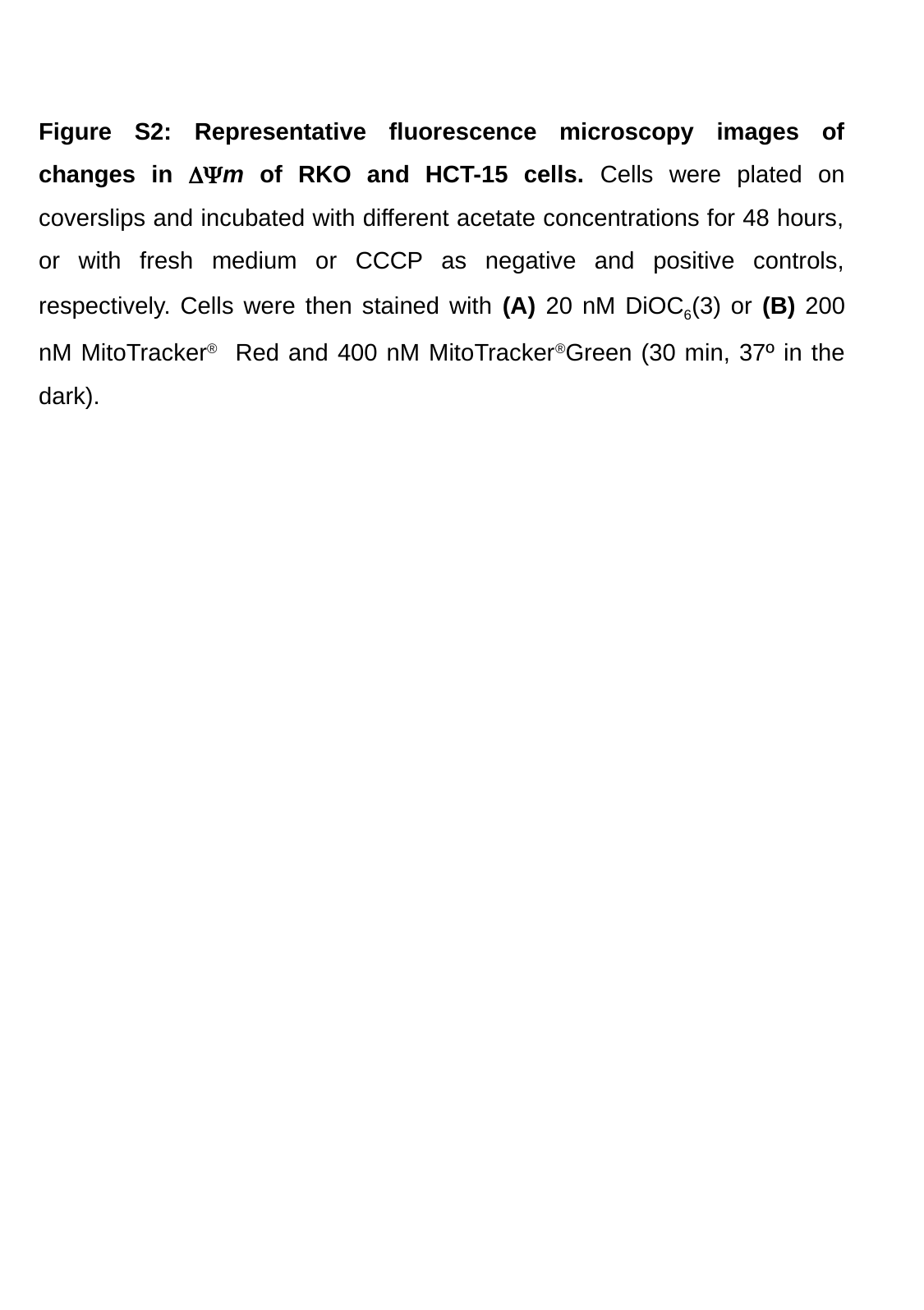

Figure S2: Representative fluorescence microscopy images of changes in m of RKO and HCT-15 cells. Cells were plated on coverslips and incubated with different acetate concentrations for 48 hours, or with fresh medium or CCCP as negative and positive controls, respectively. Cells were then stained with (A) 20 nM DiOC6(3) or (B) 200 nM MitoTracker® Red and 400 nM MitoTracker®Green (30 min, 37º in the dark).

## Slide 4
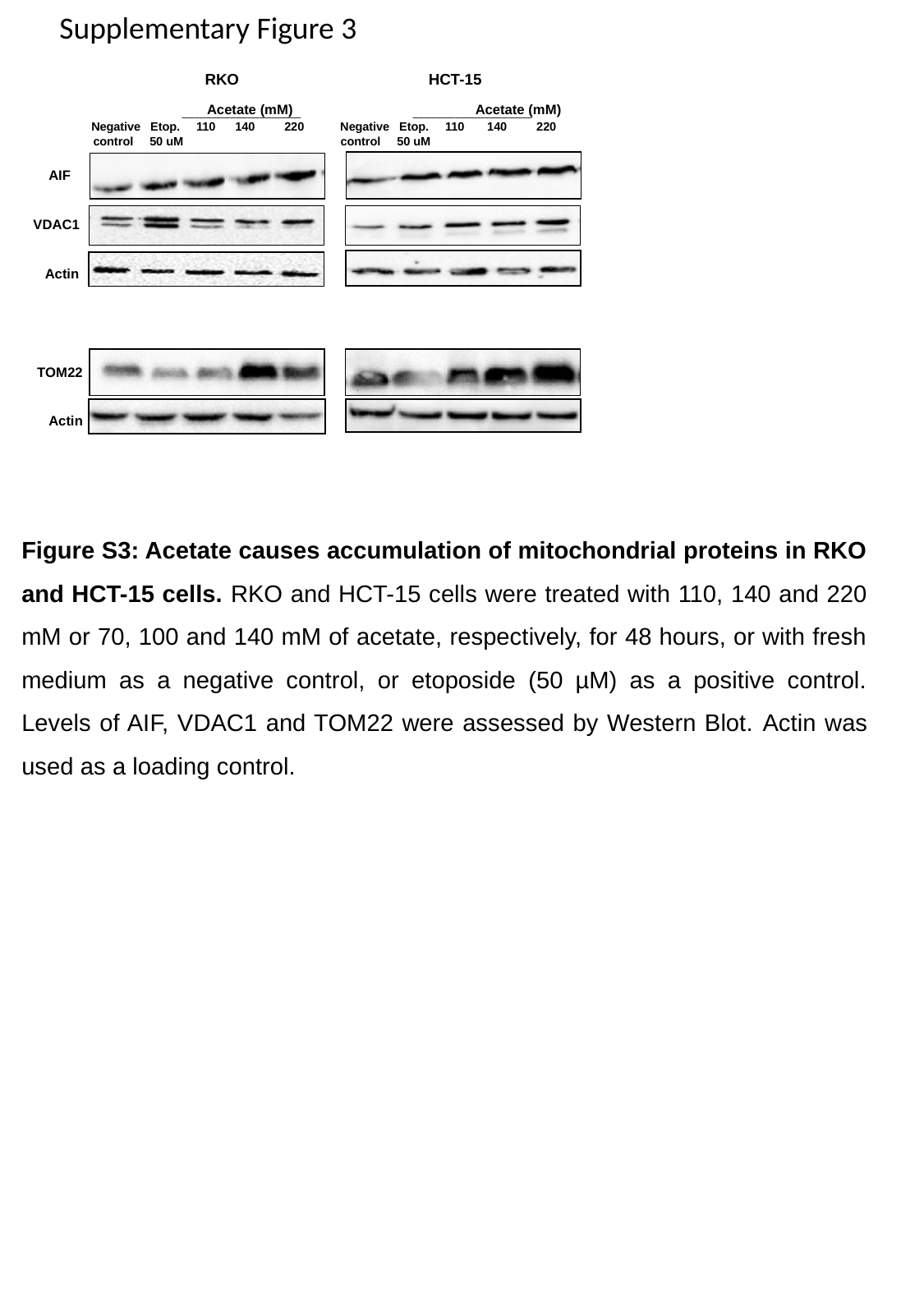

Supplementary Figure 3
 RKO HCT-15
 Acetate (mM) Acetate (mM)
 ____________________ ____________________
 Negative Etop. 110 140 220 Negative Etop. 110 140 220
 control 50 uM control 50 uM
 AIF
 VDAC1
 Actin
 TOM22
 Actin
Figure S3: Acetate causes accumulation of mitochondrial proteins in RKO and HCT-15 cells. RKO and HCT-15 cells were treated with 110, 140 and 220 mM or 70, 100 and 140 mM of acetate, respectively, for 48 hours, or with fresh medium as a negative control, or etoposide (50 µM) as a positive control. Levels of AIF, VDAC1 and TOM22 were assessed by Western Blot. Actin was used as a loading control.

## Slide 5
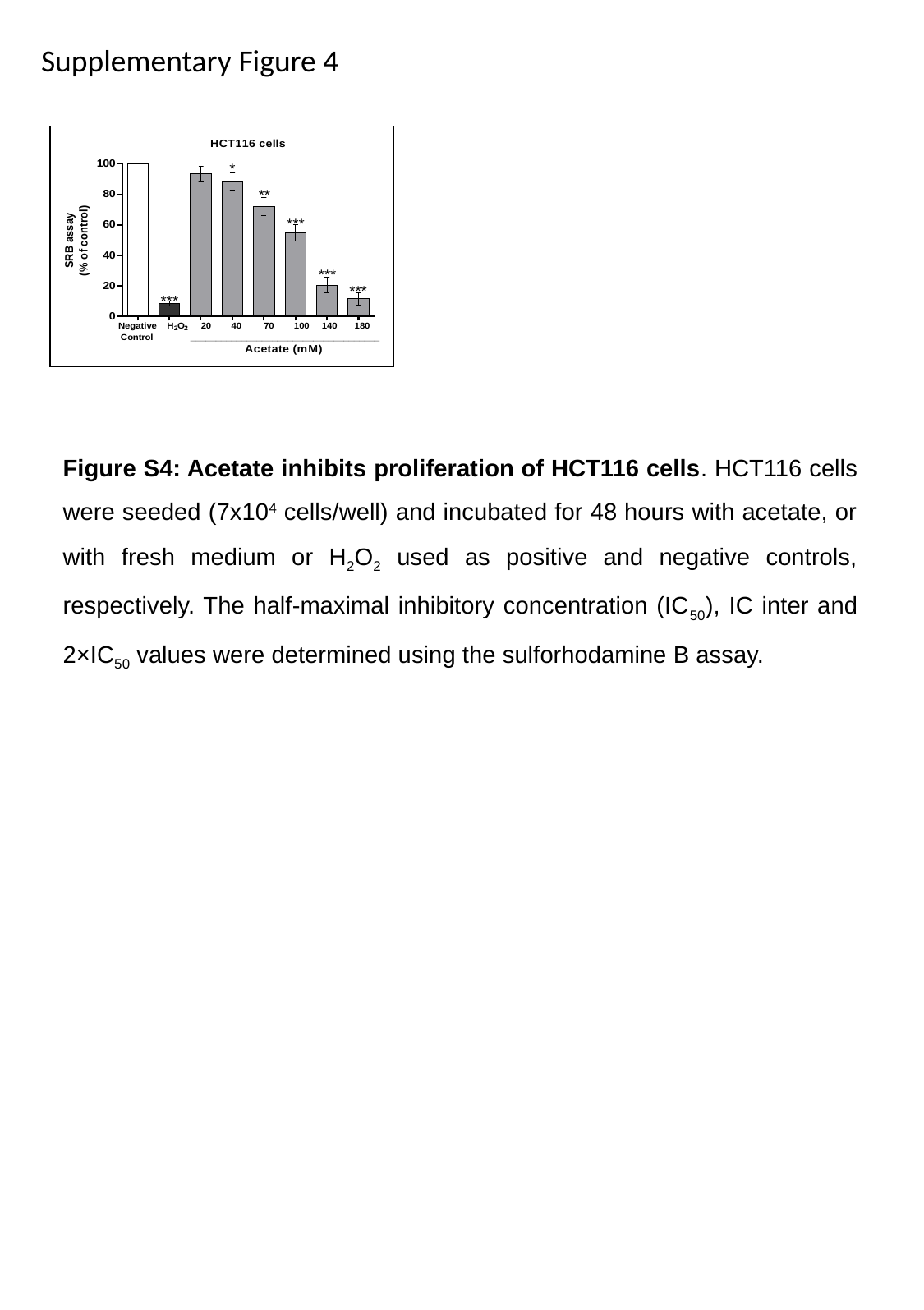

Supplementary Figure 4
Figure S4: Acetate inhibits proliferation of HCT116 cells. HCT116 cells were seeded (7x10^4^ cells/well) and incubated for 48 hours with acetate, or with fresh medium or H2O2 used as positive and negative controls, respectively. The half-maximal inhibitory concentration (IC50), IC inter and 2×IC50 values were determined using the sulforhodamine B assay.
